# Underprescription of epinephrine auto-injectors in food-allergic patients at high risk for anaphylaxis in primary care

**DOI:** 10.1186/2045-7022-5-S3-O26

**Published:** 2015-03-30

**Authors:** Jacquelien Saleh-Langenberg, Anthony E J Dubois, Feikje Groenhof, Thys Van der Molen, Bertine M J Flokstra-de Blok

**Affiliations:** 1 Department of Pediatric Pulmonology and Pediatric Allergy, University of Groningen, University Medical Centre Groningen, Groningen, The Netherlands; 2 GRIAC Research Institute, University of Groningen, University Medical Centre Groningen, Groningen, The Netherlands; 3 Department of General Practice, University of Groningen, University Medical Centre Groningen, Groningen, The Netherlands

## Background

General practitioners (GPs) play an important role in diagnosing and treating food-allergic patients. Previous studies have shown that many high risk food-allergic patients do not have an epinephrine auto-injector (EAI) and that GPs are not always knowledgeable about these patients. However, there are currently no data as to whether GPs prescribe EAIs to high risk food-allergic patients presenting to primary care practices. Therefore, the aim of this study was to obtain information about EAI prescriptions by GPs to food-allergic patients at high risk for anaphylaxis in the Netherlands.

## Methods

Patients aged 12-23 who consulted their GP for allergic symptoms were identified in a primary care database (2001-2012). Allergic symptoms were defined as ICPC-codes A12 (allergy), T04/T05 (feeding problem infant/child/adult) and EAI was defined as ATC-group C01CA24 (epinephrine). Patients were classified as probably or unlikely to be food-allergic. A risk factor based protocol was used to identify probably food-allergic patients at high risk for anaphylaxis and to assess the need for an EAI.

## Results

Out of 1314 patients who consulted their GP for allergic symptoms from 2001-2012, 148 patients (11.3%) consulted their GP for allergic symptoms due to food. Eighty patients were excluded from analyses due to incomplete data. Therefore, 68 patients were eligible for analysis. Thirty-four (50%) of these patients were classified as probably food-allergic and mostly reported symptoms from nuts (44.1%). Twenty-seven were considered high risk patients and candidates for an EAI. Importantly, only 10 (37%) of them had actually been prescribed an EAI.

## Conclusions

Although previous studies have shown that some high risk food-allergic patients do not seek medical care, this study shows that even those that do visit their GPs are often not prescribed an EAI. This shows that previously identified low rates of EAI ownership are at least partly due to failure by GPs to prescribe this medication to patients for whom it would be appropriate to do so. These data suggest that opportunities exist to improve the quality of care for high risk food-allergic patients in primary care.

